# Evaluation of medication adherence methods in the treatment of malaria in Rwandan infants

**DOI:** 10.1186/1475-2875-9-206

**Published:** 2010-07-16

**Authors:** Marc Twagirumukiza, Pierre Claver Kayumba, Jan G Kips, Bernard Vrijens, Robert Vander Stichele, Chris Vervaet, Jean Paul Remon, M Luc Van Bortel

**Affiliations:** 1 Heymans Institute of Pharmacology, Ghent University, Ghent, Belgium; 2 Department of Internal Medicine, University Hospital, Butare, Rwanda; 3 Laboratory of Pharmaceutical technology, Faculty of Pharmaceutical Sciences, Ghent University, Ghent, Belgium; 4 Department of Pharmacy, Faculty of Medicine, Butare, Rwanda; 5 Department of Biostatistics and Medical Informatics, University of Liège, Liège, Belgium

## Abstract

**Objectives:**

To compare three methods for evaluating treatment adherence in a 7-day controlled treatment period for malaria in children in Rwanda.

**Methods:**

Fifty-six children (< 5 years) with malaria were recruited at the University Hospital of Butare, Rwanda. Patients were treated with quinine sulfate, taste-masked, pellets during seven days: three days in hospital (in-patient) followed by a four-day out-patient period. Three methods to evaluate medication adherence among patients were compared: manual pill count of returned tablets, patient self-report and electronic pill-box monitoring. These pill-boxes were equipped with a microchip registering date and time of every opening. Medication adherence was defined as the proportion of prescribed doses taken. The inter-dose intervals were analysed as well.

**Results:**

Medication adherence data were available for 54 of the 56 patients. Manual pill count and patient self-report yielded a medication adherence of 100% for the in- and out-patient treatment periods. Based on electronic pill-box monitoring, medication adherence during the seven-day treatment period was 90.5 ± 8.3%. Based on electronic pill-box monitoring inpatient medication adherence (99.3 ± 2.7%) was markedly higher (p < 0.03) than out-patient adherence (82.7 ± 14.7%), showing a clear difference between health workers' and consumers' medication adherence.

**Conclusion:**

Health workers' medication adherence was good. However, a significant lower medication adherence was observed for consumers' adherence in the outpatient setting. This was only detected by electronic pill-box monitoring. Therefore, this latter method is more accurate than the two other methods used in this study.

## Background

Malaria is one of the world's most deadly diseases[1] with approximately 881,000 deaths every year, and nine out of ten deaths occurring in sub-Saharan Africa[1]. Additionally, 85% of all malaria-related deaths are recorded in children under five years of age.

The effective control and treatment of malaria presents enormous challenges, especially in sub-Saharan Africa where access to medicines and health care is limited, and multiple cases of treatment failure have been reported[2]. Poor adherence to anti-malarial treatment, from both health workers and consumers, may importantly contribute to treatment failure. Poor treatment adherence leads to sub-therapeutic drug concentrations in the body, which will not eradicate all parasites and allow growth of resistant parasites. Drug concentrations in the body can become subtherapeutic either by missed doses or by not timely taken doses. Hence, when assessing antimalarial treatment adherence it is also important to assess the timing of dose intake.

However, the evaluation of medication adherence in daily practice or in research remains challenging[3,4], in particular in the developing settings where malaria is pandemic[5,6]. Some studies have used quantitative methods such as patient and clinician reports, but questions can be raised about the reliability of these methods[7,8]. Patient self-reports have the disadvantage that they might be influenced by the desire of the patient to comply, or his/her denial of the illness[4], whereas information obtained through clinician reports could be biased by the interest the clinician may have in the patient's treatment[9,10]. Therefore, a more objective and exact alternative is provided by electronic pill-box monitoring of medication adherence. Bottle caps with built-in microchips allow to record the date and time of every opening of the medication bottle. This method has proven its value in a variety of populations with medical disorders[4,10-15], but only rarely in sub-Saharan Africa[16].

The objective of this study was twofold. Firstly, to compare three methods for evaluating adherence to an anti-malarial treatment with quinine pellets during a 7-day controlled treatment in Rwandan children. The three methods of adherence assessment were manual pill count, self-report and electronic pill-box monitoring. Secondly, since the treatment consisted of an inpatient and an outpatient period, health workers' adherence (inpatient period) was compared to consumers' adherence (outpatient period).

## Methods

### Study population

The study took place at the Rwandan University Hospital of Butare ("Centre Hospitalier Universitaire de Butare") from July to August 2006 and involved parents/guardians of 56 children less than five years of age with mild diagnosed malaria treated with taste-masked quinine sulfate pellets[17]. Among them 54 children provided exploitable data. The population sample complies with WHO guidelines[18].

### Study design

A seven day treatment regimen started with three days of full hospitalization. The full treatment consisted of 21 doses with 10 doses in hospital and 11 in the outpatient setting. Doses were calculated according to body weight. In the hospital, the drug was given by trial nurses three times daily at fixed time points: 6:00 am, 2:00 pm and 10:00 pm. In case of vomiting within half an hour after dosing, the dose was repeated and the nurse was instructed to notify this in the adverse event form. The children were discharged on the fourth day, after blood samples were taken for pharmacokinetic analysis (reported elsewhere) [17]. Whilst the discharge day was shared between the in- and outpatient setting, day five to seven were fully spent in the outpatient setting.

Upon discharge, parents/guardians were given the appropriate number of doses to complete the seven day regimen and were instructed on dosing frequency and time. This study was approved by the Ethics Committees of both Ghent University and the Ministry of Health in Rwanda.

Taste-masked quinine sulfate pellets were developed and provided by Ghent University, faculty of pharmacy, laboratory of pharmaceutical technology, and details about this formulation were reported elsewhere[17]. Every dose was individually packed in capsules and all doses per patient were delivered in labeled electronically monitored pill-boxes. Each pill-box was equipped with a microchip in its lid, registering the date and time of each opening (Medication Event Monitoring System (MEMS^®^), Aardex Corporation, Geneva, Switzerland). The parents/guardians and nurses were not aware of this recording system. However, they were instructed not to abuse the opening/closing of the pill-boxes and to keep them out of reach of children.

### Medication adherence assessment

Medication adherence was assessed in three different ways. The first method was manual pill count. For the hospitalization period, 12 doses were put in a labeled pillbox for every child by a physician and handed over to the nurse in charge. At the end of the hospitalization period, the physician counted again and noted down the exact number of capsules remaining in the pillbox for every child (this should be 2 or 3 (in 8 children) if the child did not vomit). Consequently, the physician refilled each box with 11 doses. The boxes were handed over to the parent/guardian for the ambulatory treatment. In the outpatient period, parents/guardians were asked to come to the hospital for extra pills if needed. This could be necessary for replacing vomited doses or other lost doses. All transport fees were reimbursed in such case. At the end of treatment, parents/guardians were asked to return the pill-boxes and the number of capsules in every pill-box was counted again.

A second method assessed medication adherence via self-report of the nurses and parents/guardians. This was done using a questionnaire filled out by the trial nurses at the end of hospitalization and the treatment period, respectively. Nurses and Parents/guardians were asked at which time the children had taken their medication during hospitalization and the three ambulatory days, and how often they had opened the box. They were also asked for the number of times they had opened the pill-boxes and not taken medication, and the number of times they had taken medication from other sources than the received pill-boxes. This questionnaire was tested and validated in a pilot study (involving four children for a 7-days treatment) before the present study.

Electronic pill-box monitoring was used as a third way of assessing medication adherence. Each opening was considered as a single dose intake. After the seven days treatment period, electronic pill-box monitoring data were downloaded to a personal computer using dedicated software (PowerView version 3.3.3 software - Aardex Corp, Geneva, Switzerland).

### Analysis of medication adherence

Since a standard definition and grades of good medication adherence for malaria treatment are lacking, medication adherence was analyzed according to five different definitions, which consider a patient as adherent to the treatment when:

(A) at least 70% of all doses are taken

(B) at least 80% of all doses are taken

(C) At least 90% of all doses are taken

(D) at least 80% of all doses are taken within four hours around due time (delayed/advanced for two hours)

(E) at least 80% of all doses are taken within two hours around due time (delayed/advanced for one hour)

Definitions D and E were only used for the electronic pill-box monitoring method, as the two other methods did not reveal reliable data on the timing of the dose intake. Medication adherence persistence in this study was not addressed, because of the short treatment period.

### Statistical analysis

The comparison between inpatient and outpatient data were done by chi-squared proportions' comparison test. The significance level was set at a p-value < 0.05. Data are expressed as percentages, means and standard deviations and/or 95% confidence intervals. Comparison of the 3 methods using Kappa statistics or correlation was not possible because the manual pill count and self report results were constant (adherence of 100%). For the same reason, statistics related to the interdependence of successive doses (or intervals between doses), were less informative and are skipped in the manuscript.

## Results

### Comparison between the medication adherence assessment methods over the full 7-day treatment period

Medication adherence data from 54 of the 56 patients (96.4%) were recovered after completion of the seven days treatment. Two parents did not return their electronically monitored pill-box. Medication adherence assessed by the three methods are shown in Table 1. According to manual pill count, the medication adherence was 100% (for definitions A to C). In fact, no parent or guardian returned any capsule at the final visit. Also for the self-report method, adherence was 100% for definitions A to C. All parents/guardians stated to have exactly followed the given instructions, and they confirmed that they had been compliant with the time schedule. Using the electronic pill-box monitoring, medication adherence varied largely according to the used definition (Table 1). None of the children got 100% medication adherence. The strictest definition (E) yielded 33.3% of adherence, whereas the least strict definition (A) yields an adherence of 83.3% throughout the seven day treatment regimen.

**Table 1:** Medication adherence according to different adherence definitions, assessed by electronic pill-box monitoring.

Adherence definition	Inpatient* (10 doses)	Outpatient* (11 doses)	Whole treatment* (21 doses)#
(A) > 70% doses taken	54/54 (100.0%)	45/54 (83.3%)	45/54 (83.3%)

(B) > 80% doses taken	54/54 (100.0%)	42/54(77.8%)	42/54(77.8%)

(C) > 90% doses taken	52/54 (96.3%)	25/54 (46.3%)	23/54 (42.6%)

(D) > 80% of doses within four hours around due time (delayed/advanced of two hours)	53/54 (98.1%)	38/54 (70.4%)	32/54 (59.3%)

(E) > 80% of doses within two hours around due time (delayed/advanced of one hour)	39/54(72.2%)	22/54(40.7%)	18/54(33.3%)

*number of patients (and percentage) adherent according to adherence definition

# Overall adherence can be lower than the minimum of in- and outpatient adherence when the non-adherent children in the inpatient setting are different from those in the outpatient setting.

**Figure 1:**
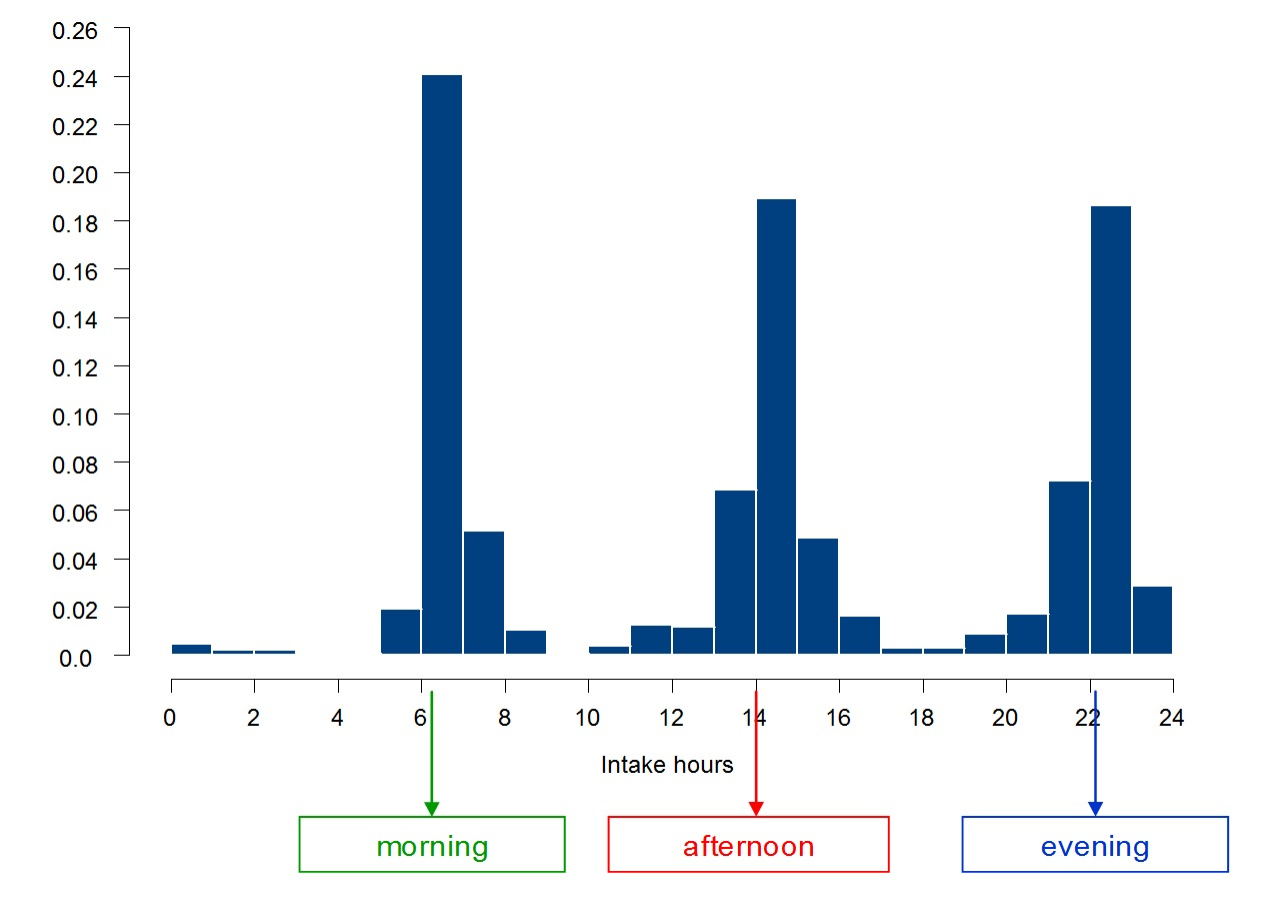
**Recorded clock time of opening of the electronically monitored pill-boxes**. The figure 1 shows the percentage of total openings recorded with electronically monitored pill-boxes by clock time.

An overall view on the dosing times according to the electronic pill-box monitoring records shows that the morning intake ranged from 5:00 am to 8:00 am (mode = 6:00 am), the noon intake from 10:00 am to 5:00 pm (mode = 2:00 pm) and the evening intake ranged from 7:00 pm to 3:00 am (mode = 10:00 pm) (Figure 1).

Four parents/guardians confirmed to have opened the electronically monitored pill-box accidentally. Three guardians reported that their child vomited and that the dose was replaced within 30 minutes. However, this has not been confirmed by electronic pillbox monitoring. None of parents/guardians had taken medication from other sources than the received pill-boxes, nor did any parent/guardian return to the hospital for extra pills.

### Health workers' versus consumers' adherence

Manual pill count and self-report did not show any differences between health workers' (inpatient) and consumers' (outpatient) adherence, as adherence was 100% throughout the treatment for these two methods. Electronic pill-box monitoring however shows a much lower compliance during the outpatient than during the inpatient period (Table 1). When looking at the number of missed doses rather than at medication adherence (Table 2), this difference is even more pronounced. In the hospital, only two out of the 429 monitored doses (0.5%) were missed, versus 72 of the 568 (12.7%) monitored doses in the outpatient setting (Table 3).

**Table 2:** Number of missed doses, as measured by electronic pill-box monitoring.

Settings	Total number of missed doses	range of missed doses per patient	Medication adherence expressed as % of doses taken (95% CI)
**Inpatient** **10 doses per patient**	2 doses	0 to 2 doses	99.3 (96.6-100.0)

**Outpatient** **11 doses per patient**	72 doses	0 to 6 doses	82.7 (68.0-97.4)

**In total**	74 doses	0 to 6 doses	90.5 (82.2-98.8)

**Table 3:** Doses taken in time: comparison between hospitalized versus outpatient setting, as measured by electronic pill-box monitoring.

The time when the dose has been taken, compared with the prescribed time.		hospitalisation period (429 doses)		outpatient period (568 doses)		p-value
			
		Number of doses	%	Number of doses	%	
doses taken too late	more than 1 hour	40	9.3	89	15.7	0.013*
	
	more than 2 hours	2	0.5	23	4.0	0.002*

doses taken too early	more than 1 hour	11	2.6	49	8.6	0.001*
	
	more than 2 hours	5	1.2	26	4.6	0.003*

*Statistically significant between hospitalization and the outpatient period

Additionally, electronic pill box monitoring allows to assess the time of dosing throughout the treatment. The two missed doses in the hospital were evening doses. In the outpatient setting, 12% of the morning doses, 18% of the noon doses and 9% of the evening doses were missed. Table 3 lists the number of doses taken more than 1 or 2 hours too early or too late, which were significantly higher in the outpatient than in the inpatient setting. As a consequence, the same difference between inpatient and outpatient setting was found for deviations in the interdose interval (Table 4).

**Table 4:** Interdose interval comparison between hospitalized versus outpatient setting, as measured by electronic pill-box monitoring.

The interval between doses		hospitalisation period (373 intervals)		outpatient period (462 intervals)		p-value
			
		Number of doses	%	Number of doses	%	
interdose	longer than 9 hours	17	4.0	115	20.2	< 0.001*
	
	Longer than 10 hours	6	1.4	57	10.0	< 0.001*

interdose	shorter than 7 hours	35	8.2	93	16.4	0.001*
	
	shorter than 6 hours	14	3.3	38	6.7	0.023*

*Statistically significant between hospitalization and the outpatient period

## Discussion

In this study manual pill count, self-reports and electronic pill-box monitoring were used to assess the adherence of health workers and parents/guardians of 56 children to a seven-day malaria treatment with taste-masked quinine sulfate pellets. To the authors' knowledge, this is the first study to compare these three methods of medication adherence assessment in a short-period treatment in an African setting. In addition, although medication adherence during hospitalization and in an ambulatory setting have been widely discussed[5,16,19-21], the authors are not aware of data comparing hospital to the outpatient malaria medication adherence in the same study elsewhere in sub-Saharan settings. Hence, this study is the first to report on the difference between health workers' (inpatient) and consumers' (outpatient) medication adherence in the African setting. The study also introduces the importance of assessing the inter-dose intervals when studying anti-malarial medication adherence.

Results from the pillbox monitoring revealed a clearly lower adherence in the outpatient setting (consumers' adherence) than in the inpatient setting (health workers' adherence) (Table 1). The number of missed doses and time of dose intake variability was higher during the outpatient period (Tables 2 &3). In outpatients, the evening doses were the most adhered, followed by the morning doses. This finding, if confirmed by other studies, may imply that once daily dosing treatments should better be given in the evening than in the morning hours. The difference in medication adherence between the health workers and the consumers is probably due to the hospital setting and the health workers' professional competence, but may also be influenced by the child's condition, which was improved at hospital discharge.

The very similar medication adherence estimates obtained from self-report and manual pill count, which are markedly higher than the estimate obtained by electronic pill-box monitoring (Table 1) is in line with literature data, and has been attributed to the fact that patients have some desire to present themselves as compliant[22]. The fact that no guardian/parents returned any left-over drug, even if not all doses were taken according to the electronic pill-box monitoring, may be compatible with this desire to be compliant.

Electronic pillbox monitoring allows a more objective and accurate monitoring of medication adherence, showing clearly lower levels of adherence. Although electronic pillbox monitoring is accepted to yield a fairly objective quantitative estimate, independent from many variables that could bias the estimates and may be less intrusive than direct observation, this method is not free from limitations either. The boxes can be opened without medication being taken, thus causing an overestimation of the medication adherence. Medication adherence may also be underestimated by electronic pill-box monitoring. This happens when multiple doses are taken out at one pill-box opening[9,23]. Therefore, it should be advised to combine electronic pillbox monitoring with interrogation on accidental opening and taking multiple doses at 1 pillbox opening. In the Rwandan setting, where parents/guardians use to spend the all journey in the field-mainly in subsistence agriculture, one could assume that the pillbox was not always carried with them and multiple doses were taken out at home as a provision. However, when inspecting the dispersion of box-openings in time in the outpatient setting, boxes were not opened more often in the morning than they were in the afternoon or in the evening. If patients wish to deceive the method, they must open the electronically monitored pillbox on the same time as if they should actually take the pills[11,24]. Although electronic pillbox monitoring may be a reference tool of medication adherence evaluation, its high cost should be emphasized. This may limit their use to specific studies such as the evaluation of interventions intended to improve treatment adherence.

Furthermore, some parents/guardians reported that their child vomited and that the dose was replaced within 30 minutes. This means the child should have received more than the prescribed number of eleven doses. Since each parent/guardian received eleven doses upon discharge from the hospital and not one parent/guardian came back to the hospital for refill, this causes an inconsistency in the medication adherence assessment that could not be solved via either adherence monitoring method.

The variability in adherence is mainly driven by the variability in the ambulatory setting since at the hospital the adherence was nearly 100%. This high hospital adherence resulted in an effective decrease in parasitaemia and appropriate quinine plasma levels at discharge from the hospital[17] and may explain why despite the altered consumers' adherence no treatment failure has been reported in the present study.

## Conclusion

Electronic pillbox monitoring proves superior to pill count and patient self-report in medication adherence assessment, which suggests that also in short treatment interventions in the sub-Saharan setting it could be used as a reference tool for treatment adherence assessment.

The present study also shows a markedly lower adherence in the outpatient setting (consumers' adherence) than in the inpatient setting (health workers' adherence). This lower medication adherence applies to not only the number of doses, but also to the right timing of dosing. In outpatients, the evening doses were the most adhered, followed by the morning doses.

The present study also shows that an appropriate in hospital treatment period may decrease treatment failure and probably also drug resistance.

## Competing interests

The authors declare that they have no competing interests.

## Authors' contributions

MT, PCK, CV, JPR, RVS and LVB were involved in the conception, planning of the work, results interpretation, drafting and edition of the manuscript. JK and BV were involved in data handling and verification. BV, JK, RVS and LVB were involved in data analysis and interpretation, critical revision of the manuscript particularly advice on treatment adherence evaluation methods. All authors were involved in reading, commenting/editing and the approval process of final version of the manuscript.
